# Effectiveness of exercise prescription variables to reduce fall risk among older adults: a meta-analysis

**DOI:** 10.1186/s11556-025-00374-x

**Published:** 2025-05-13

**Authors:** Tian-Rui Zhu, Hong-Qi Xu, Jin-Peng Wei, He-Long Quan, Xue-Jiao Han, Tian-Xiang Li, Ji-Peng Shi

**Affiliations:** Northeast Normal University, Changchun, China

**Keywords:** Fall Risk, Elderly, Exercise Intervention, Meta-analysis

## Abstract

**Objective:**

This meta-analysis explored the relationship between various exercise prescription variables and their effects on fall risk reduction in older adults, enabling the selection of targeted and evidence-based intervention prescription variables tailored to individual risk-assessment results.

**Method:**

Databases including PubMed, Embase, Web of Science, and the Cochrane Library were systematically searched for randomized controlled trials that investigated the impact of exercise intervention on fall prevention. Study quality was assessed using the Cochrane risk-of-bias tool. Meta-analyses, subgroup analyses, sensitivity analyses, and assessments of publication bias were performed using Stata 16.0.

**Results:**

A total of 43 articles comprising 51 studies and involving 2,743 participants were included. The results indicated significant improvements in fall risk assessment indices due to Mind–body Exercise (MBE), Multi-component Physical Activity (MCPA), and Muscle-strengthening Activity(MSA). Subgroup analyses revealed differential optimal type, cycle (week), frequency (day/week), and session time (minutes) across assessment tools, such as the unipedal stance test with eyes open (MCPA, < 8, 3, 45 ≤ Time < 60), functional reach (MCPA, < 8, < 3, ≥ 60), the “get-up and go” test (MSA, ≥ 24, < 3, 30 ≤ Time < 45), Berg balance scale (MBE, 8 ≤ Time < 12, 3, 30 ≤ Time < 45), Five stands sit-to-stand (MCPA, ≥ 24, > 3, 30 ≤ Time < 45), the 30-s chair-stand test (MSA, 12 ≤ Time, < 3, 45 ≤ Time < 60), short physical performance battery (MCPA, 12 ≤ Time < 24, < 3, ≥ 60), and Falls Efficacy Scale-International (MBE, 8 ≤ Time < 12, < 3, 45 ≤ Time < 60).

**Conclusion:**

The findings suggest that prescription variables combining MCPA and MBE, ≥ 8-week programs, and ≥ 30-min sessions, effectively reduce fall risk through concurrent enhancement of balance, strength, and self-efficacy; their integration into community-based protocols with individualized resistance-balance combinations optimizes functional outcomes in older adults.

## Introduction

A fall refers to the event of an individual suddenly landing on the floor, ground, or lower level [1]. Estimates based on data from the World Health Organization suggest that falls—as the second leading cause of unintentional injury deaths worldwide—resulted in 684,000 individuals dying, with adults 60 years and older suffering the greatest number of fatal falls [2]. Fall-related injuries are not only responsible for long-term pain, motor dysfunction, disability, and even death but may also lead to the development of depression or anxiety due to a subsequent fear of falling [3], which causes tremendous physical and psychological harm to older adults and economic burden to family and society [4]. Research has shown that multiple factors can increase the risk of falls in older adults [5], which increases the difficulty of risk screening and disease prevention. Previous systematic reviews and network meta-analyses have extensively compared the efficacy of various fall prevention factors across heterogeneous older populations, have failed to establish clear, targeted guidelines for community-dwelling populations [6–8]. Studies have reported that ineffective or harmful exercises incorporated similar prescription variables [9], which not only suggests similar components may lack cross-contextual effectiveness but also highlights the methodological challenges arising from inconsistencies in outcome measurements, heterogeneous population characteristics, and the absence of evidence-based intervention when developing exercise prescriptions for older adults. In the rapidly aging global population, the prevention of falls for older people is a very important question worldwide.

Delbaere et al. [10]—with a decision tree model—have shown that the loss of balance is a key predictor of falls. The critical role of balance training and the necessity of sustained, long-term exercise programs to effectively reduce fall risk had confirmed [11]. Thus, exercise intervention is also considered the most appropriate and economical intervention approach at group level [8, 12, 13]. Current meta-analyses suggest that exercise interventions can help prevent falls in the older population. Multiple types of exercise produce the best results, followed by Tai Chi [7]. Despite the growing body of literature supporting exercise interventions for fall prevention in older adults, there is still a lack of clarity about the optimal combination of exercise regimes. Currently, most studies are unable to produce a structured, individualized exercise plan (including form, cycle, frequency, and time) based on the physical fitness test results of the individuals in the manner of exercise prescription. While the World Guidelines for Falls Prevention and Management for Older Adults (hereafter referred to as the guidelines) emphasize that effective fall prevention programs require individualized exercises with regular review and progression, the recommendation for older adults at lower risk to participate in 150–300 min of moderate-intensity or 75–150 min of vigorous-intensity activity per week in a safe condition remains insufficiently precise [14]. This limitation stems from the absence of standardized protocols for dynamic dose adjustments based on quantitative monitoring of individual functional progression (e.g., balance ability, muscle strength, and fall efficacy). However, few studies have examined the dose–response relationship between cycle, frequency, and duration of exercise regimes and their ability to reduce the risk of falling. This meta-analysis aimed to address these gaps by analyzing different exercise modalities and providing evidence-based recommendations for tailored interventions.

## Method

The study was registered on the Preferred Reporting Items for Systematic Review and Meta-Analyses (PRISMA) and conducted according to the *PRISMA 2020 Statement: An Updated Guideline for Reporting Systematic Reviews* [15].

### Search strategy

A comprehensive literature search was performed using PubMed, Embase, Web of Science, and the Cochrane Library from their inception to November 15, 2024. Search terms were grouped into four key concepts: (1) falls, (2) older adults, (3) exercise, and (4) randomized controlled trials (RCTs). To ensure the inclusion of high-quality RCTs, search filters developed by McMaster University's Health Information Research Unit were applied [16]. The search strategy combined both Medical subject headings terms and free-text words. Studies were included based on predefined criteria, which are detailed in Table 1.

**Table 1:** Inclusion criteria and exclusion criteria

Criteria	Type	Definition
Inclusion	Patients	Adults over 60 years of age, living independently in the nursing home or community
	Intervention	The experimental group only received one or more exercise interventions; the control group maintained normal daily activity
	Comparison	The control group maintained normal daily activity, received conventional nursing, received health education, or performed sham exercises with no gain
	Outcomes	1. Balance ability: the unipedal stance test with eyes open [17], functional reach [18], Berg balance scale [19], and “get-up and go” test [20]; 2. Lower limb muscle strength: five stands sit-to-stand [21], the 30-s chair-stand test [22], and short physical performance battery [23]; 3. Fall-efficacy: Falls Efficacy Scale-International (FES-I) [24]
	Study	RCTs (Randomized controlled trials)
Exclusion		1. The adults had serious diseases, cognitive impairment, or care needs or were living with assistive devices 2. Did not provide a definitive exercise intervention program 3. The data were not presented in means ± standard deviations descriptive form

### Screening process

All retrieved articles were imported into Endnote X9 for systematic management. The articles were screened by two independent researchers based on the inclusion and exclusion criteria. Following a full-text assessment of retrieved articles, two researchers conducted reference list screening to identify potentially includable studies. Before consensus was reached, the agreement between them was quantified using Cohen's kappa coefficient. Any disagreements were resolved by a third reviewer.

### Quality assessment

The Cochrane Handbook [24] for Systematic Reviews of Interventions (version 6.4) was used to assess the risk of bias, and Review Manager software 5.3 was used to conduct the assessment. The risk of bias across studies was evaluated in terms of randomization, allocation concealment, blinding, and completeness of outcome data.

### Data extraction

Two independent researchers screened titles/abstracts of all identified articles. Abstracts meeting inclusion criteria were retrieved as full-text articles. Full texts were then assessed by the same reviewers. Discrepancies were adjudicated by a third reviewer until consensus was achieved, with corresponding authors contacted for additional data when necessary. Data extraction encompassed pre-post intervention quantitative data from both experimental and sham-control conditions, extracted from text and tables in each included study. Two researchers independently extracted relevant data from the selected articles.

The extracted information included study characteristics (author, publication year, sample size, country), participant demographics (age, gender), intervention details (exercise type, cycle, frequency, duration), and outcome measures related to fall risk and physical performance. For the simplification of the analysis and result application, the exercise types were divided into MBE (Controlled movement practices through mindful motor control and breath awareness), MSA (Targeted exercises applying resistance to induce neuromuscular adaptations for strength enhancement), and MCPA (Integrated interventions combining ≥ 2 training domains to optimize functional capacity) according to previously published studies [25–27].

### Statistical analysis

The meta-analysis was conducted using Stata software (Version 16.0SE; Stata Corp, College Station, TX, USA). Heterogeneity across studies was evaluated using Cochran’s Q test and the I^2^ statistic (1–50% = low, 50–75% = moderate, 75–100% = high heterogeneity), with a *p*-value < 0.1 or I^2^ > 50% indicating significant heterogeneity. Random-effects models were employed in cases of above moderate heterogeneity, while fixed-effects models were applied when heterogeneity was minimal. Hedges’ g and 95% credible intervals (CrIs) were used to assess the credibility of the estimates. Publication bias was tested using Egger’s test and a funnel plot. Subgroup analyses were performed to investigate potential sources of heterogeneity and exercise prescription variables’ effects on functional capacity related to fall risk.

## Results

### Study selection

**Fig. 1:**
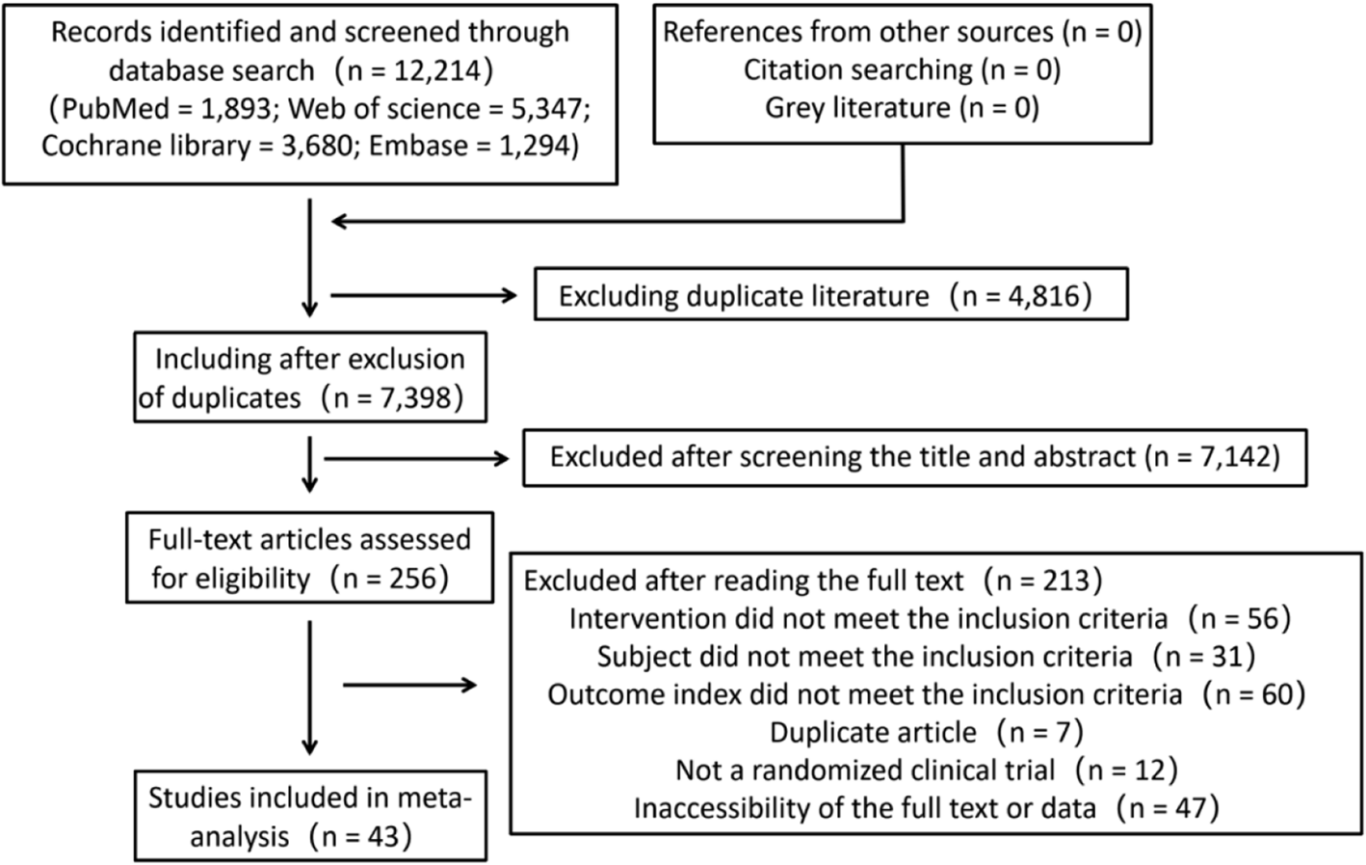
PRISMA flow chart of the study selection process

A total of 12,214 records were retrieved through preliminary searching. According to the above inclusion and exclusion criteria, two researchers independently screened and extracted literature by reading the title, abstract, and full text. Finally, 43 studies were included (Fig. 1).

### Characteristics of the included studies

In total, 43 articles (1 in Korean and 42 in English) were included in the literature review, including 51 studies (if an article included more than one intervention, then each exercise intervention was split into the relevant study). The study sample included a total of 2,743 subjects, with the exercise group consisting of 1,503 and the control group consisting of 1,240 subjects. The age range of the participants was 60–75 years, and they lived in the community. Inter-rater reliability between the two independent researchers was assessed using Cohen's kappa of 0.87.

The exercise interventions included MBE (*n* = 29), MSA (*n* = 7), and MCPA (*n* = 15). Of the included studies, because only three [28–30] used walking as the intervention for the purpose of improving balance and cognitive ability, these interventions were grouped together as an MBE. The characteristics of each sample are shown in Table 2.

**Table 2:** Characteristics of the included studies

Author	Country	Experimental Group					Control group	Primary outcome indicator
		Sample size	Intervention component	Cycles (week)	Frequency (times/week)	Time (min)	Sample size	
Arkkukangas 2019 (1) [31]	Sweden	61	Otago exercise program (OEP)—MCPA	12	3	30	28	⑧
Arkkukangas 2019 (2) [31]	Sweden	58	OEP combined with Motivational Interviewing (OEP + MI)—MCPA	12	3	30	28	⑧
Arrieta 2018 [32]	Spain	57	Warm-up (5 min); Strength training (40%−60%1RM/65–70% 1RM, 25 min); Balance training (10 min); Walking retraining (5 min)—MCPA	12	2	45	55	⑤, ⑦, ⑧
Benavent 2015 [33]	Spain	28	Warm up; Strengthening exercises (ankle cuff weights, starting at 0.5 kg); Balance exercises; Walk (usual pace, 10 min); Cool down (10 min)—MCPA	16	3	45	23	①, ④, ⑤, ⑧
Brüll 2023 (1) [28]	Germany	23	Warm-up (5 min); Perturbation blocks (4 min*four perturbation); Cool down (3 min)—MBE	6	3	25	10	④, ⑦
Brüll 2023 (2) [28]	Germany	27	Warm-up (5 min); Circuit training (unstable devices: standing, lunges, jumping, five stations devices * 3 min); Cool down (3 min)—MBE	6	3	25	11	④, ⑦
Chang 2011 [34]	Korea	10	Warm-up (5-10 min); Main exercises (upper extremity, lower extremity muscular strength exercises, balancing exercises, 20–30 min); Cool down (5-10 min)—MBE	4	> 3	35	8	⑤
Chewning 2019 [35]	United States	94	Opening (5–10 min); Tai Chi; Warm-ups and basic moves instruction (20–30 min); Informal teatime (10 min); Group home practice enhancement activities (20–30 min); TCF short form instruction (10–25 min); Closing (5 min)—MBE	6	2	90	103	④, ⑦
Donatoni 2022 [36]	Ireland	17	Pilates intervention overview (warm-up, mat pilates with accessories, cool down, 10repetitions * 2-3 sets)—MCPA	12	2	60	31	③, ④, ⑨
Fakhro 2019 [37]	Lebanon	30	Warm-up (5 min); Balance training using the Wii Fit games (soccer heading + table tilt, 30 min); Cool down (5 min)—MBE	8	3	40	30	④
Ferraro 2019 [38]	United Kingdom	23	IMT (equivalent to ~ 50% of baseline MIP, 30 breaths); Sham-IMT (corresponding to ~ 15% baseline MIP, 60 slow breaths)—MSA	8	7	30	23	④, ⑥
Franco 2020 [39]	Brazil	35	Senior dance classes (moderate-level intensity)—MBE	12	2	60	36	②, ⑥
Gabizon 2016 [40]	Israel	34	Lying exercises (thera-band); Sitting exercises; Sitting exercises; Thera-band exercises—MCPA	12	3	60	44	⑤
Granacher 2021 [41]	Germany	27	A lifestyle exercise program that included balance exercises conducted during the daily tooth brushing routine (3 min * twice)—MBE	8	7	6	24	④, ⑤, ⑥
Hartmann 2009 (1) [42]	Switzerland	28	Warm-up (10 min); Aerobic exercise (15 min); Progressive resistance strength training (12 repetitions, 2–3 sets); Foot gymnastics (5 min); Stretching; Relaxation exercises (10 min); Home-program (2-min Warm up, 4-min foot gymnastics, 4-min stretching)—MCPA	12	2	50	7	⑨
Hartmann 2009 (2) [42]	Switzerland	28	Warm-up (10 min); Aerobic exercise (15 min); Progressive resistance strength training (12 repetitions, 2–3 sets); Stretching; Relaxation exercises (10 min)—MSA	12	2	40	7	⑨
Hewitt 2018 [43]	Australia	113	Resistance; Weight-bearing balance; Functional group exercise sessions (10–15 repetitions, 2–3 sets, 30 min)—MCPA	25	2	60	108	⑧, ⑨
Hirase 2015 (1) [44]	Japan	32	Warm-up (10 min); Balance training (double-stance standing, one-leg standing, neck hyperextension, free-leg swinging, heel and toe raises, neck and trunk rotation, touching the floor, walking in place, sideways walking, and forward walking,40 min); Cool down (10 min)—MBE	16	1	60	15	④, ⑥
Hirase 2015 (2) [44]	Japan	31	Warm-up(10 min);Balance training(double-stance standing, one-leg standing, neck hyperextension, free-leg swinging, heel and toe raises, neck and trunk rotation, touching the floor, walking in place, sideways walking, and forward walking,40 min);Cool down(10 min)—MBE	16	1	60	15	④, ⑥
Hosseini 2018 [45]	Iran	30	Warm up (5 min); Tai Chi (35 min); Cool down (5 min)—MBE	8	2	55	30	④, ⑨
Ing 2024 [46]	Malaysia	26	Warm up; Resistance exercise using strap-on weights; Balance training; Square-stepping; Cool down and backward chaining to train for getting up from the floor—MCPA	24	2	75	26	①, ④, ⑥, ⑦
Jin 2012 [47]	Korea	17	Warm up;Static exercise (7 min * twice); Dynamic exercise (10 min); Progressive balance exercise (10 min); Cool down—MBE	4	2	60	18	①, ④, ③
Jung 2020 [48]	Japan	18	Movement of the lower extremity and spine to a greater extent; Put additional weight—MCPA	24	1	60	15	③, ④, ⑥
Kwon 2011 [49]	Korea	32	Warm-up (10 min); Walking exercise (20 min); Resistance training with elastic band (8–10/10–15 repetitions, 20 min); Education sessions (30-60 min); Cool-down (10 min)—MSA	12	1	60	21	④, ⑧
Lai 2013 [50]	China	15	Xavix Measured Step System (XMSS)—MBE	6	3	30	15	④, ⑤
Lee 2017 [51]	Korea	27	Stretching (3 min); Warm-up (5 min); Main exercise (static exercise, dynamic exercise, progressive balance exercises, 40 min); Cool down (10 min)—MBE	4	2	60	27	①, ③, ④, ⑤
Lee 2023 [52]	Korea	28	Warm-up (warm-up exercise consisted of stretching using the Ring Fit program and a leg massage using a massage ball, 10 min); Exercise (the participants performed yoga to increase balance, and leg and abdominal exercises to strengthen the lower-extremity muscles, 30 min); Cool down (stretching and breathing exercises, 10 min)—MBE	8	3	50	29	①, ③, ④, ⑤, ⑥
Machacova 2015 [53]	Czech	27	Warm-up (10 min); Main dance-based exercises (foxtrot, waltz, cha-cha-cha, cancan, and so on,40 min); Cool down (10 min)—MBE	12	1	60	25	⑦
Manor 2014 [54]	United States	26	Raising the power; Withdraw and push; Grasp the sparrow's tail; Brush knee twist step; Wave hand like clouds(20 min * three times weekly)—MBE	12	2	60	28	④, ⑤, ⑧
Naczk 2020 [55]	Poland	10	12 sets of exercises (upper and lower extremities, 10 kg/20 kg, 3 sets per muscle group)—MSA	6	2	30	10	⑦
Oh 2020 [56]	Korea	11	Relax their bodies and meditate (10 min); Motor imagery training (20 min); Task-Oriented training (20 min)—MBE	6	3	40	12	④, ⑤
Ohtake 2013 [57]	Japan	92	Six types of stretching exercises (15 s * 5 sets); Six types of muscle strength training (3 s * 5 sets); Two types of balance training (3 s * 5 sets); Toe stretching; A resistance band was used for muscle strength training—MCPA	8	2	25	74	③, ④
Pepera 2022 [58]	Greece	20	Warm-up (10 min); Main component (30 min); Cool down (10 min)—MCPA	8	2	45	20	⑥
Pirouzi 2014 [29]	Iran	14	Warm-up (5 min); Forward treadmill training (10 min); Backward treadmill training (10 min); Cool down exercises (5 min)—MBE	4	3	30	15	⑤
Roller 2017 [59]	United States	27	The older adults walked in pairs or trios/an aerobic activity in an indoor space—MBE	10	1	45	28	④, ⑤
Sadaqa 2024 [60]	Hungary	12	Walking + map reading—MBE	12	2	50	12	③, ④, ⑧
Sales 2016 [61]	Finland	27	Pilates (progressive resistance of 2–4 springs, 8–10 repetitions)—MCPA	18	2	60	21	①, ④, ⑦
Schilling 2009 [62]	-	10	Warm-up (range of motion exercises + light walking, 5 min); Progressive static and dynamic balance exercises (13–15 repetitions maximum, one or two sets,10 min); Strength exercises (15-20 min); Aerobic exercises (15-20 min); Cool down exercises (5 min).—MCPA	5	3	30	9	④
Schlicht 2001 [63]	United States	11	Warm-up (5-10 min); Core training (push-ups and taps on the platform, modified pull-ups and gangway, balance stool, calf raises + finger steps, sit to stand and round snake pipe, ramp + net + climb through and sharp snake pipe, balance beam and hip extension, steps and screws/turners,step-ups and hip abduction, 45-75 min); Cool down (5-10 min)—MBE	8	3	45	11	②, ⑥
Sedaghati 2022 [64]	Iran	14	Body squat; Leg circles; Extended reach; Standing balance; Sit-to-stand; Split squats; Forward trunk lean; Standing balance; Body squat; Trunk rotation; Diagonal trunk lean; Extended reach (3repetitions, 2–3 sets)—MBE	8	3	60	14	④, ⑤, ⑧
Sitthiracha 2021 [65]	Thailand	30	Leg extension; Hip adduction; Hip abduction; Gluteal press; Leg press and ankle extension (75% 1RM, 10 repetitions, 2 sets)—MSA	8	5	40	30	①, ②, ④, ⑥, ⑨
Timon 2021 (1) [66]	Spain	18	Strength static balance; Dual-task; Corrective posture (first month); Strength; Dynamic balance; Dual-task;Corrective posture (second month)—MCPA	24	3	45	9	①, ⑦, ⑨
Timon 2021 (2) [66]	Spain	17	Warm up; Main part (PSME); Cool down—MBE	24	3	45	10	①, ⑦, ⑨
Printes 2024 (1) [30]	Brazil	24	Warm-up (10 min); Main part (strength training, 12-15repetitions * three sets, 6-8RPE, 4 kg-6 kg, 30 min); Cool down (5 min); 459 m—MSA	24	2	60	13	④
Printes 2024 (2) [30]	Brazil	23	Warm-up (10 min); Main part (strength training, 12-15repetitions * three sets, 6-8RPE, 4 kg-6 kg, 30 min); Cool down (5 min); 2000 m, FIO2 = 16.1%—MSA	24	2	60	12	④
Ullmann 2010 [67]	United States	25	Sitting; Reaching; Walking; Turning; Transfers (lying to sitting, sitting to standing, and vice versa); Relaxation—MBE	5	3	60	22	④
Whyatt 2015 [68]	United Kingdom	40	Apple Catch;Bubble Pop;Avoid the Shark;Smart Shrimp(30 min)—MBE	5	2	30	42	⑤
Witte 2017 (1) [69]	Germany	28	Warm-up (10-15 min); Specific training (various stances, arm techniques during standing, simple attack and defense exercises, 40-45 min); Cool down (5 min)—MBE	20	2	60	13	⑥
Witte 2017 (2) [69]	Germany	23	Warm-up (10-15 min); Specific training (elements of gymnastics, running exercises, practices with a ball and other hand devices, strengthening exercises based on manuals, 40-45 min); Cool down (5 min)—MBE	20	2	60	13	⑥
Wu 2021 (1) [70]	United States	12	Begin Tai Chi; Part the horse’s mane; Brush knee and push; Cloud hands; Open and close; Part the grass; Single whip; Finish Tai Chi.—MBE	12	3	60	5	①, ④, ⑦
Wu 2021 (2) [70]	United States	13	Begin Tai Chi; Roll the ball; Kick with the heel; Repulse the monkey; Gather the earth’s qi; White crane spread wing; Fairy weaves the shuttle; Finish Tai Chi.—MBE	12	3	60	5	①, ④, ⑦

Primary outcome indicator: ① the unipedal stance test with eyes open; ② the unipedal stance test with eyes closed; ③ functional reach; ④ “get-up and go” test: ⑤ berg balance scale; ⑥five stands sit-to-stand; ⑦ the 30-s chair-stand test; ⑧ short physical performance battery; ⑨ falls efficacy scale-international. MCPA, multi-component physical activity; MSA, muscle-strengthening activity; MBE, mind–body exercise

The MBE was dominated by traditional Chinese martial arts or balance and functional training, accounting for 56.86% (*n* = 29). The intervention cycle ranged from 4 to 25 weeks, of which 12–24 accounted for 39.22% (*n* = 20). Interventions less than three times a week had the highest percentage of 54.9%. Each exercise time of more than 60 min had the highest percentage of 45.1%. A summary of exercise prescription variables’ characteristics is shown in Table 3.

**Table 3:** Summary of exercise prescription variables characteristics

Prescription Variables	Grouping criteria	Proportion
Form	Multi-component physical activity	29.41% (*n* = 15)
	Muscle–strengthening activity	13.73% (*n* = 7)
	Mind–body exercise	56.86% (*n* = 29)
Cycle (week)	< 8	25.49% (*n* = 13)
	8 ≤ Time < 12	21.57% (*n* = 11)
	12 ≤ Time < 24	39.22% (*n* = 20)
	≥ 24	13.73% (*n* = 7)
Frequency (day/week)	< 3	54.9% (*n* = 28)
	3	37.25% (*n* = 19)
	> 3	7.84% (*n* = 4)
Time (minutes)	< 30 min	7.84% (*n* = 4)
	30 ≤ Time < 45 min	25.49% (*n* = 13)
	45 ≤ Time < 60 min	21.57% (*n* = 11)
	≥ 60 min	45.1% (*n* = 23)

### Quality of the included literature and publication bias

**Fig. 2:**
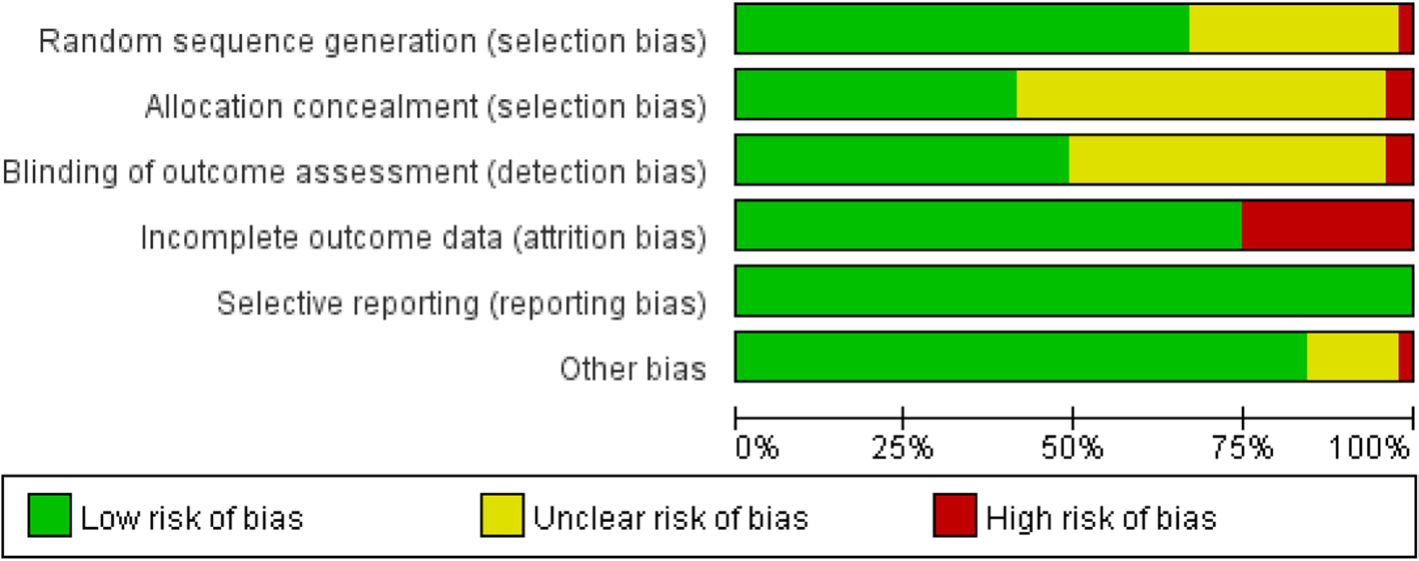
Summary of risk of bias for each item presented as a percentage across all included studies

The quality assessment data are summarized and presented in Fig. 2. In total, 32 of the 51 studies applied random allocation and were illustrated. Additionally, there was some difficulty in the blinded manner due to the characteristics of the exercise interventions; therefore, 25 studies applied the blind method and 14 studies explicitly reported the allocation concealment. A total of 30 studies reported complete outcome data.

**Fig. 3:**
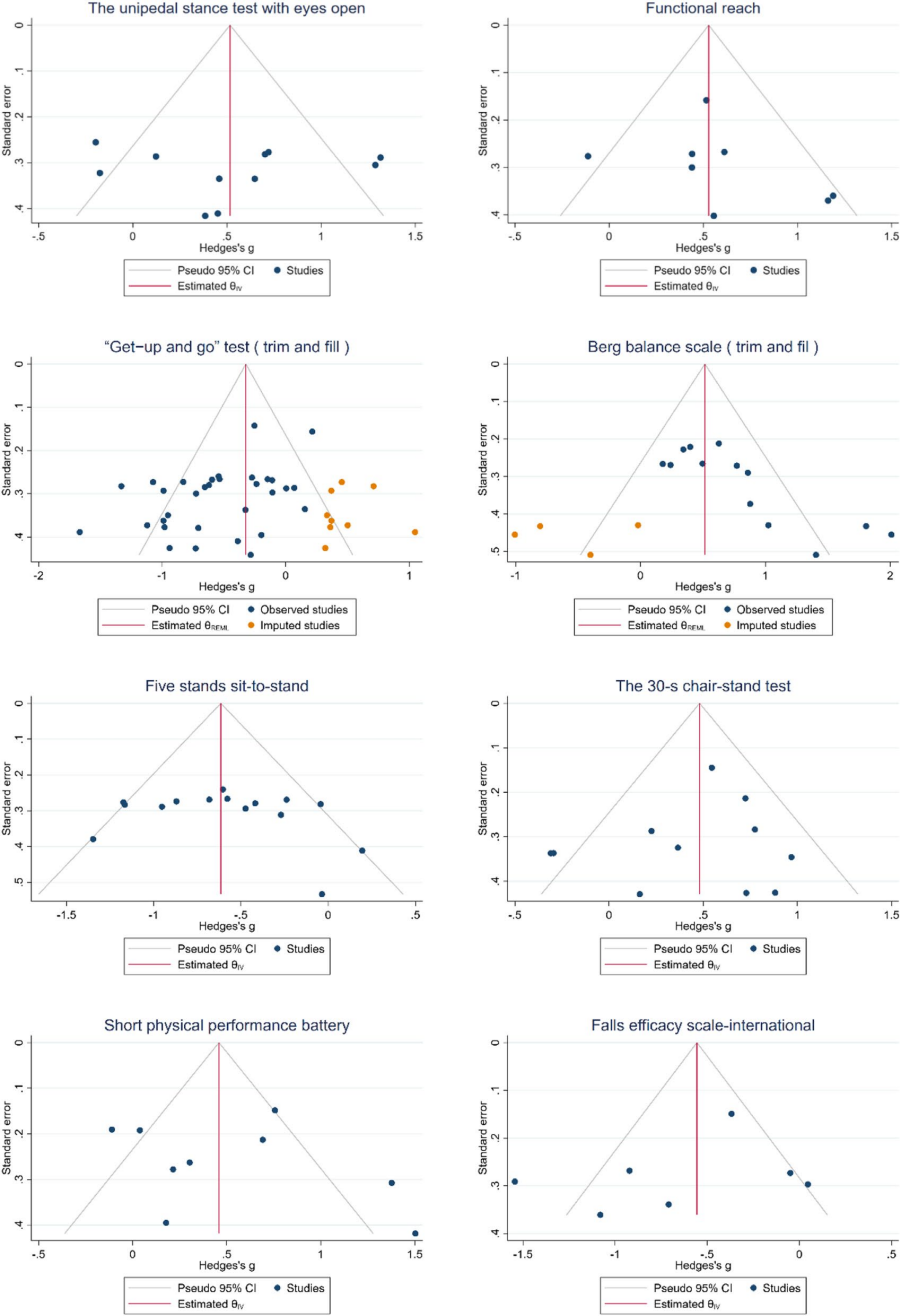
Funnel plot for publication bias

Additionally, the risk of publication bias was assessed using the funnel plot (Fig. 3) and Egger’s test (Table 4). Funnel plot asymmetry and Egger's test revealed potential publication bias only in the “Get − up and go” test (t = −3.17, *p* < 0.05) and Berg balance scale (t = 4.30, *p* < 0.05) subgroups. Trim and fill method adjustment demonstrated marginally lower effect sizes (adjusted g = −0.32, 95% CI −0.50 to −0.14 and 0.52, 95% CI 0.17 to 0.86) compared to observed values (g =  − 0.52, 95% CI −0.68 to −0.37 and 0.74, 95% CI 0.47 to 1.01) for these subgroups, as visually substantiated in Fig. 3.

**Table 4:** Total effect of exercise intervention on fall risk in older adults

Subgroup/Variables	Number of included studies	Heterogeneity test		Effect model	Result of Meta-analysis		Egger's test
		Q(*P*)	I^2^/%		Hedges’s g with 95% CI	P	
The unipedal stance test with eyes close	3	*P* = 0.01	73.52%	Random	0.36 [− 0.29, 1.01]	*P* = 0.28	-
The unipedal stance test with eyes open	11	*P* < 0.01	64.99%	Random	0.52 [0.21,0.83]	*P* < 0.01	*P* = 0.87
Functional reach	8	*P* = 0.10	41.64%	Fixed	0.53 [0.34, 0.71]	*P* < 0.01	-
“Get − up and go” test	32	*P* < 0.01	59.23%	Random	− 0.52 [− 0.68, − 0.37]	*P* < 0.01	*P* < 0.05
Berg balance scale	13	*P* < 0.01	62.29%	Random	0.74 [0.47, 1.01]	*P* < 0.01	*P* < 0.05
Five stands sit-to-stand	15	*P* = 0.02	48.14%	Fixed	− 0.61 [− 0.76, − 0.47]	*P* < 0.01	*P* = 0.47
The 30-s chair-stand test	11	*P* = 0.05	44.61%	Fixed	0.48 [0.32, 0.64]	*P* < 0.01	*P* = 0.52
Short physical performance battery	9	*P* < 0.01	79.66%	Random	0.51 [0.16, 0.86]	*P* < 0.01	-
Falls efficacy scale-international	7	*P* < 0.01	78.01%	Random	− 0.64 [− 1.07, − 0.22]	*P* = 0.01	-

### Meta-analysis

**Fig. 4:**
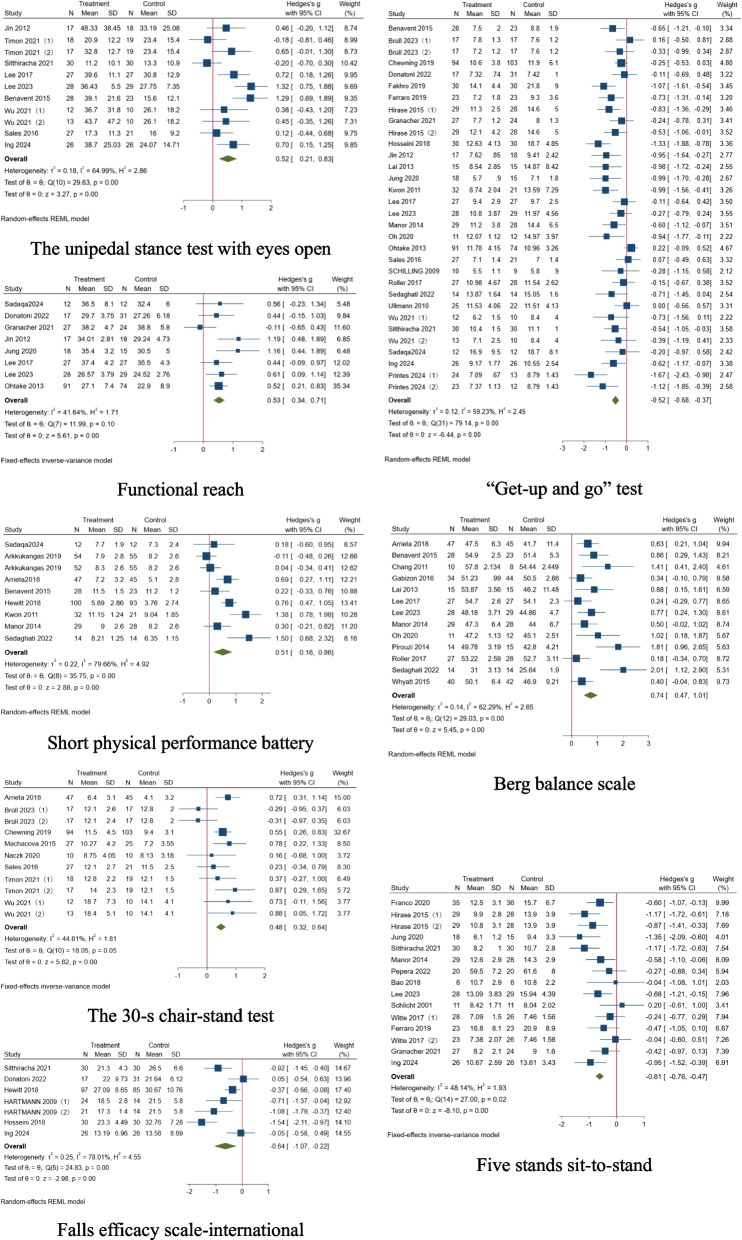
Forest plot of the meta-analysis regarding the effect of exercise intervention on fall risk in older adults

The effect of the exercise intervention on balance in older adults was calculated by comparing their scores for the pre-and post-intervention unipedal stance tests with closed eyes, the unipedal stance test with eyes open, functional reach, “get-up and go” test, Berg balance scale, five stands sit-to-stand, the 30-s chair-stand test, short physical performance battery, and FES-I (Fig. 4).

As shown in Table 4, 3, 11, 8, 32, 13, 15, 11, 9, and 7 studies were included in the above subgroup, which shows some heterogeneity (I^2^ = 73.52%, 64.99%, 41.64%, 59.23%, 62.29%, 48.14%, 44.61%, 79.66%, and 78.01%). Among the 9 subgroups analyzed, 33.33% demonstrated low heterogeneity, 44.44% moderate heterogeneity, and 22.22% high heterogeneity, with Cochran's Q values ranging from 18.05 to 79.14 (all *p* ≤ 0.05).

In addition to the unipedal stance test with eyes closed (*p* = 0.28), the pooled effect size of each research using the random-effect model was statistically significant, suggesting that exercise intervention significantly improved the test scores for balance in older adults (*p* < 0.01).

### Meta-regression and subgroup analysis

Meta-regression and subgroup analysis were performed for different exercise prescription variables that could be included (Table 5). Meta-regression demonstrated statistically significant associations between session time of MCPA and Short physical performance battery improvements, with a coefficient of 0.51 (95% CI 0.02 to 1.00,* p* < 0.05). While long-term exercise efficacy in reducing fall risk has been substantiated, subgroup analyses stratified by prescription variables were implemented to address remaining heterogeneity sources and optimize the effectiveness of exercise protocol.

**Table 5:** The results of meta-regression analysis

Screening tools\Exercise types	Covariates	Coef	p	95% CI
The unipedal stance test with eyes open				
MBE I^2^ = 63.93%, Random g = 0.97, 95% CI 0.57 to 1.38	Cycle	−0.18	0.68	−1.44—1.08
	Frequency	0.06	0.94	−2.51—2.64
	Time	0.21	0.8	−2.16—2.58
Functional reach				
MCPA I^2^ = 0%, Fixed g = 0.58, 95% CI 0.34 to 0.82	Cycle	0.71	0.38	−5.33—6.75
	Frequency			
	Time	−0.27	0.47	−3.33—2.79
MBE I^2^ = 67.13%, Random g = 0.50, 95% CI 0.01 to 1.00	Cycle	0.2	0.86	−10.93—11.32
	Frequency			
	Time	0.36	0.49	−4.16—4.88
“Get-up and go” test				
MCPA I^2^ = 58.07%, Random g = −0.35, 95% CI −0.65 to −0.04	Cycle	−0.25	0.24	−0.75—0.25
	Frequency	−0.44	0.18	−1.19—0.31
	Time	−0.12	0.34	−0.43—0.19
MBE I^2^ = 58.78%, Random g = −0.56, 95% CI −0.76 to −0.36	Cycle	−0.21	0.06	−0.44—0.01
	Frequency	0.26	0.21	−0.18—0.71
	Time	0.15	0.26	−0.12—0.43
Berg balance scale				
MCPA I^2^ = 79.47%, Random g = 0.73, 95% CI 0.20 to 1.25	Cycle	−0.57	0.68	−14.01—12.86
	Frequency	0.16	0.93	−17.52—17.85
	Time	0.56	0.76	−17.41—18.55
MBE I^2^ = 53.86%, Random g = 0.77, 95% CI 0.44 to 1.10	Cycle	0.04	0.83	−0.43—0.5
	Frequency	0.51	0.09	−0.13—1.15
	Time	−0.1	0.59	−0.56—0.37
Five stands sit-to-stand				
MCPA I^2^ = 62.53%, Random g = −0.83, 95% CI −1.43 to −0.23	Cycle	−0.41	0.28	−2.87—2.04
	Frequency	omitted		
	Time	omitted		
MBE I^2^ = 45.63%, Fixed g = −0.62, 95% CI −0.80 to −0.45	Cycle	−0.65	0.29	−2.04—0.73
	Frequency	−1.38	0.08	−3—0.25
	Time	−0.76	0.1	−1.72—0.21
The 30-s chair-stand test				
MSA I^2^ = 22.89%, Fixed g = 0.54, 95% CI 0.13 to 0.94	Cycle	0.16	0.5	−1.91—2.24
	Frequency	omitted		
	Time	omitted		
MBE I^2^ = 63.34%, Random g = 0.09, 95% CI −0.39 to 0.56	Cycle	−0.02	0.88	−0.42—0.37
	Frequency	0.3	0.47	−0.85—1.46
	Time	0.38	0.09	−0.1—1.46
Short physical performance battery				
MCPA I^2^ = 79.66%, Random g = 0.51, 95% CI 0.16 to 0.86	Cycle	−0.39	0.17	−1.07—0.3
	Frequency	−0.17	0.57	−1.04—0.69
	Time	0.51	0.04	0.02—1.00
Falls efficacy scale-international				
MCPA I^2^ = 44.55%, Fixed g = −0.27, 95% CI −0.49 to −0.05	Cycle	−0.34	0.48	−4.46—3.78
	Frequency	omitted		
	Time	0.76	0.34	−4.97—6.48

*Abbreviations*: *MCPA* multi-component physical activity, *MSA* muscle-strengthening activity, *MBE* mind–body exercise, *Random* random-effect model, *Fixed* fixed-effect model. I^2^, Heterogeneity test statistic; g, Pooled effect size (Hedges'g) for exercise-type subgroup analysis; 95% CI, 95% confidence interval

To explore the potential source of heterogeneity, we sequentially excluded the included studies and found no differences in the overall outcomes. As shown in Table 6, a subgroup analysis of potential moderating variables in each group was carried out to further explore the sources of heterogeneity and the effects of the exercise prescription variables on functional capacity related to fall risk. Among the 82 subgroups analyzed, 47.56% (*n* = 39) demonstrated low heterogeneity, 42.68% (*n* = 35) moderate heterogeneity, and 9.76% (*n* = 8) high heterogeneity. The meta-analysis results showed significant improvements in balance and strength across multiple indices, with effect sizes indicating clinically meaningful improvements. The different components of intervention programs (type, duration, frequency, and session length of exercise) had varying dose–response relationships with the results of fall risk screening tests in older adults. For instance, the subgroup analysis results indicated that the unipedal stance test with eyes open for older adults engaged in MCPA (g = 0.97, 95% CI 0.57–1.38,* p* < 0.05) was larger than others, indicating superior efficacy compared to MBE and MSA for enhancing static balance capacity, which directly correlated with reduced fall risk. Notably, different trends were observed regarding the optimal exercise prescription components (e.g., frequency, session duration) across metrics evaluating balance, muscular strength, and mental efficacy in older adults, necessitating evidence-based selection of optimal components to ensure personalized intervention protocols.

**Table 6:** Results of each subgroup analysis

Subgroup/Variables		Grouping criteria	Number of included studies	Heterogeneity test		Effect model	Result of Meta-analysis	
				Q(*P*)	I^2^/%		Hedges’s g with 95% CI	P
The unipedal stance test with eyes open								
Intervention form	MCPA		2	*P* = 0.16	49.78%	Fixed	0.97 [0.57, 1.38]	*P* < 0.01
	MSA		2	*P* = 0.08	68.06%	Random	0.23 [− 0.58, 1.04]	*P* = 0.57
	MBE		7	*P* = 0.01	63.93%	Random	0.46 [0.07, 0.85]	*P* = 0.02
Cycle (week)	< 8		2	*P* = 0.55	0%	Fixed	0.61 [0.20, 1.03]	*P* < 0.01
	8 ≤ Time < 12		2	*P* < 0.01	93.5%	Random	0.55 [− 0.93, 2.04]	*P* = 0.46
	12 ≤ Time < 24		4	*P* = 0.04	61.32%	Random	0.57 [0.03, 1.12]	*P* = 0.04
	≥ 24		3	*P* = 0.09	59.04%	Random	0.40 [− 0.15, 0.95]	*P* = 0.16
Frequency (day/week)	< 3		4	*P* = 0.41	0%	Fixed	0.51 [0.22, 0.80]	*P* < 0.01
	3		6	*P* < 0.01	68.3%	Random	0.68 [0.19, 1.16]	*P* < 0.01
	> 3		1	-	-	-	-	-
Time (minutes)	< 30		-	-	-	-	-	-
	30 ≤ Time < 45		1	-	-	-	-	-
	45 ≤ Time < 60		4	*P* < 0.01	80.17%	Random	0.78 [0.09, 1.47]	*P* = 0.03
	≥ 60		6	*P* = 0.70	0%	Fixed	0.49 [0.23, 0.75]	*P* < 0.01
Functional reach								
Intervention form	MCPA		4	*P* = 0.41	0%	Fixed	0.58 [0.34, 0.82]	*P* < 0.01
	MSA		-	-	-	-	-	-
	MBE		4	*P* = 0.03	67.13%	Random	0.50 [0.01, 1.00]	*P* < 0.05
Cycle (week)	< 8		2	*P* = 0.1	63.78%	Random	0.78 [0.05, 1.51]	*P* < 0.04
	8 ≤ Time < 12		3	*P* = 0.05	57.64%	Random	0.37 [− 0.03, 0.77]	*P* = 0.1
	12 ≤ Time < 24		2	*P* = 0.82	0%	Fixed	0.48 [− 0.01, 0.95]	*P* < 0.05
	≥ 24		1	-	-	-	-	-
Frequency (day/week)	< 3		6	*P* = 0.32	15.40%	Fixed	0.61 [0.40, 0.82]	*P* < 0.01
	3		1	-	-	-	-	-
	> 3		1	-	-	-	-	-
Time (minutes)	< 30		2	*P* = 0.05	74.29%	Random	0.24 [− 0.37, 0.85]	*P* = 0.44
	30 ≤ Time < 45		-	-	-	-	-	-
	45 ≤ Time < 60		2	*P* = 0.91	0%	Fixed	0.59 [0.16, 1.03]	*P* < 0.01
	≥ 60		4	*P* = 0.17	40.78%	Fixed	0.72 [0.41, 1.03]	*P* < 0.01
“Get-up and go” test								
Intervention form	MCPA		8	*P* < 0.01	58.07%	Random	− 0.35 [− 0.65, − 0.04]	*P* < 0.05
	MSA		2	*P* = 0.53	0%	Fixed	− 0.86 [− 1.27, − 0.45]	*P* < 0.01
	MBE		22	*P* < 0.01	58.78%	Random	− 0.56 [− 0.76, − 0.36]	*P* < 0.01
Cycle (week)	< 8		9	*P* = 0.13	36.45%	Fixed	− 0.31 [− 0.49, − 0.13]	*P* < 0.01
	8 ≤ Time < 12		9	*P* < 0.01	73.92%	Random	− 0.51 [− 0.84, − 0.17]	*P* < 0.01
	12 ≤ Time < 24		10	*P* = 0.41	20.53%	Fixed	− 0.51 [− 0.70, − 0.32]	*P* < 0.01
	≥ 24		4	*P* = 0.18	38.5%	Fixed	− 1.01 [− 1.35, − 0.68]	*P* < 0.01
Frequency (day/week)	< 3		17	*P* < 0.01	73.40%	Random	− 0.56 [− 0.81, − 0.31]	*P* < 0.01
	3		12	*P* = 0.13	32.15%	Fixed	− 0.50 [− 0.69, − 0.31]	*P* < 0.01
	> 3		3	*P* = 0.47	0%	Fixed	− 0.49 [− 0.81, − 0.18]	*P* < 0.01
Time (minutes)	< 30		4	*P* = 0.32	15.06%	Fixed	0.06 [− 0.17, 0.29]	*P* = 0.61
	30 ≤ Time < 45		6	*P* = 0.59	0%	Fixed	− 0.77 [− 1.03, − 0.51]	*P* < 0.01
	45 ≤ Time < 60		5	*P* = 0.02	66.28%	Random	− 0.53 [− 0.97, − 0.09]	*P* = 0.02
	≥ 60		17	*P* < 0.01	54.57%	Random	− 0.57 [− 0.78, − 0.36]	*P* < 0.01
Berg balance scale								
Intervention form	MCPA		5	*P* < 0.01	79.47%	Random	0.73 [0.20, 1.25]	*P* < 0.01
	MSA		-	-	-	-	-	-
	MBE		8	*P* = 0.04	53.86%	Random	0.77 [0.44, 1.10]	*P* < 0.01
Cycle (week)	< 8		5	*P* = 0.02	69.36%	Random	0.79 [0.27, 1.31]	*P* < 0.01
	8 ≤ Time < 12		4	*P* < 0.01	80.30%	Random	0.89 [0.21, 1.57]	*P* < 0.01
	12 ≤ Time < 24		4	*P* = 0.28	21.5%	Fixed	0.56 [0.30, 0.81]	*P* < 0.01
	≥ 24		-	-	-	-	-	-
Frequency (day/week)	< 3		5	*P* = 0.68	0%	Fixed	0.41 [0.20, 0.63]	*P* < 0.01
	3		7	*P* = 0.01	64.85%	Random	1.02 [0.60, 1.44]	*P* < 0.01
	> 3		1	-	-	-	-	-
Time (minutes)	< 30		-	-	-	-	-	-
	30 ≤ Time < 45		5	*P* = 0.03	59.32%	Random	1.02 [0.50, 1.53]	*P* < 0.01
	45 ≤ Time < 60		4	*P* = 0.29	19.41%	Fixed	0.60 [0.35, 0.85]	*P* < 0.01
	≥ 60		4	*P* < 0.01	84.41%	Random	0.70 [− 0.02, 1.42]	*P* = 0.05
Five stands sit-to-stand								
Intervention form	MCPA		3	*P* = 0.07	62.53%	Random	− 0.83 [− 1.43, − 0.23]	*P* < 0.01
	MSA		2	*P* = 0.19	42.88%	Fixed	− 0.25 [− 0.72, 0.22]	*P* = 0.30
	MBE		10	*P* < 0.01	45.63%	Fixed	− 0.62 [− 0.80, − 0.45]	*P* = 0.06
Cycle (week)	< 8		-	-	-	-	-	-
	8 ≤ Time < 12		7	*P* = 0.1	43.39%	Fixed	− 0.53 [− 0.76, − 0.29]	*P* < 0.01
	12 ≤ Time < 24		6	*P* = 0.06	53.28%	Random	− 0.58 [− 0.90, − 0.27]	*P* < 0.01
	≥ 24		2	*P* = 0.41	0%	Fixed	− 1.10 [− 1.55, − 0.65]	*P* < 0.01
Frequency (day/week)	< 3		9	*P* = 0.03	53.01%	Random	− 0.65 [− 0.93, − 0.38]	*P* < 0.01
	3		3	*P* = 0.16	44.78%	Fixed	− 0.36 [− 0.77, 0.05]	*P* = 0.08
	> 3		3	*P* = 0.1	55.85%	Random	− 0.69 [− 1.18, − 0.21]	*P* = 0.01
Time (minutes)	< 30		1	-	-	-	-	-
	30 ≤ Time < 45		2	*P* = 0.08	66.77%	Random	− 0.83 [− 1.52, − 0.15]	*P* = 0.02
	45 ≤ Time < 60		4	*P* = 0.30	18.48%	Fixed	− 0.33 [− 0.67, 0.01]	*P* = 0.05
	≥ 60		8	*P* = 0.03	55.02%	Random	− 0.70 [− 0.99, − 0.41]	*P* < 0.01
The 30-s chair-stand test								
Intervention form	MCPA		1	-	-	-	-	-
	MSA		3	*P* = 0.27	22.89%	Fixed	0.54 [0.13, 0.94]	*P* < 0.01
	MBE		7	*P* = 0.03	58.93%	Random	0.36 [− 0.02, 0.70]	*P* = 0.04
Cycle (week)	< 8		4	*P* = 0.03	63.34%	Random	0.09 [− 0.39, 0.56]	*P* = 0.72
	8 ≤ Time < 12		-	-	-	-	-	-
	12 ≤ Time < 24		5	*P* = 0.59	0%	Fixed	0.65 [0.39, 0.90]	*P* < 0.01
	≥ 24		2	*P* = 0.20	38.07%	Fixed	0.65 [0.18, 1.11]	*P* < 0.01
Frequency (day/week)	< 3		5	*P* = 0.50	0%	Fixed	0.55 [0.36, 0.75]	*P* < 0.01
	3		6	*P* = 0.02	61.48%	Random	0.36 [− 0.11, 0.83]	*P* = 0.13
	> 3		-	-	-	-	-	-
Time (minutes)	< 30		2	*P* = 0.98	0%	Fixed	− 0.30 [− 0.77, 0.16]	*P* = 0.21
	30 ≤ Time < 45		1	-	-	-	-	-
	45 ≤ Time < 60		3	*P* = 0.43	0%	Fixed	0.69 [0.38, 1.00]	*P* < 0.01
	≥ 60		5	*P* = 0.61	0%	Fixed	0.57 [0.35, 0.78]	*P* < 0.01
Short physical performance battery								
Intervention form	MCPA		9	*P* < 0.01	79.66%	Random	0.51 [0.16, 0.86]	*P* < 0.01
	MSA		-	-	-	-	-	-
	MBE		-	-	-	-	-	-
Cycle (week)	< 8		-	-	-	-	-	-
	8 ≤ Time < 12		1	-	-	-	-	-
	12 ≤ Time < 24		7	*P* < 0.01	74.45%	Random	0.37 [0.01, 0.73]	*P* < 0.05
	≥ 24		1	-	-	-	-	-
Frequency (day/week)	< 3		5	*P* = 0.06	59.09%	Random	0.69 [0.35, 1.02]	*P* < 0.01
	3		4	*P* < 0.01	85.21%	Random	0.34 [− 0.30, 0.97]	*P* = 0.30
	> 3		-	-	-	-	-	-
Time (minutes)	< 30		-	-	-	-	-	-
	30 ≤ Time < 45		2	*P* = 0.58	0%	Fixed	− 0.04 [− 0.30, 0.23]	*P* = 0.78
	45 ≤ Time < 60		3	*P* = 0.29	18.92%	Fixed	0.46 [0.16, 0.77]	*P* < 0.01
	≥ 60		4	*P* = 0.02	74.95%	Random	0.93 [0.41, 1.45]	*P* < 0.01
Falls efficacy scale-international								
Intervention form	MCPA		4	*P* = 0.14	44.55%	Fixed	− 0.27 [− 0.49, − 0.05]	*P* < 0.05
	MSA		1	-	-	-	-	-
	MBE		2	*P* = 0.12	59.51%	Random	− 1.22 [− 1.83, 0.61]	*P* < 0.01
Cycle (week)	< 8		-	-	-	-	-	-
	8 ≤ Time < 12		2	*P* = 0.12	59.51%	Random	− 1.22 [− 1.83, − 0.61]	*P* < 0.01
	12 ≤ Time < 24		3	*P* = 0.04	67.85%	Random	− 0.56[− 1.22, 0.11]	*P* = 0.10
	≥ 24		2	*P* = 0.31	4.8%	Fixed	− 0.30 [− 0.55, − 0.04]	*P* = 0.03
Frequency (day/week)	< 3		6	*P* < 0.01	80.64%	Random	− 0.60 [− 1.09, − 0.11]	*P* = 0.02
	3		-	-	-	-	-	-
	> 3		1	-	-	-	-	-
Time (minutes)	< 30		-	-	-	-	-	-
	30 ≤ Time < 45		2	*P* = 0.73	0%	Fixed	− 0.98 [− 1.40, − 0.56]	*P* < 0.01
	45 ≤ Time < 60		2	*P* = 0.06	71.32%	Random	− 1.15 [− 1.96, − 0.33]	*P* < 0.01
	≥ 60		3	*P* = 0.17	7.27%	Fixed	− 0.24 [− 0.47, −0.04]	*P* < 0.05

*Abbreviations*: *MCPA* multi-component physical activity, *MSA* muscle-strengthening activity, *MBE* mind–body exercise, *Random* random-effect model, *Fixed* fixed-effect model

In addition, the results demonstrate that while exercise interventions and their optimal prescription components (cycle, frequency) induced statistically significant differences in “Get-up and go” test performance, the effect sizes did not reach Minimal Clinically Important Differences (MCIDs). As shown in Table 7, interventions and prescription parameters exceeded MCIDs thresholds for the Berg balance scale and Short physical performance battery. Future studies are warranted to establish validated MCIDs across functional screening tools for older adults with varying health statuses. These statistical calculations facilitate a refined evaluation of intervention efficacy.

**Table 7:** Comparison of the effect size of interventions versus fall risk with MCID values

Outcome	Total effect	The optimal prescription components				MCID
		Form	Cycle	Frequency	Time	
“Get-up and go” test	−1.38	−3.27	−1.53	−1.55	−2.80	1.6 [71]
Berg balance scale	3.11	3.82	3.12	3.73	4.11	1.9 [72]
Short physical performance battery	1.09	0.96	0.79	1.79	1.90	0.4 [71]

*Abbreviations*: *MCID* minimal clinically important difference

## Discussion

### Fall risk

This study compared the effectiveness of different exercise regimes for decreasing fall risk and identifying the dose–effect relationship of regime elements and assessment indices. The results suggest that exercise interventions can reduce the risk of falls by improving balance, lower-extremity muscle strength, and physical mobility, as well as reducing the fear of falling in older adults. More importantly, the optimal intervention protocol according to subgroup results was better for delivering targeted exercise interventions, therefore improving the fall-preventing capacity for older adults.

### Balance

Balance, as the basic ability to maintain the equilibrium of body posture, is very important to avoid falls. However, neuromuscular deficits (e.g., sarcopenia) associated with aging may lead to impaired physical performance and an increased risk for falls [73]. As the optimal and economic intervention, exercise intervention is a diverse, systematic, and organized approach at improving physical health [7, 8]. The favorable intervention effects of MCPA and MBE align with the findings of previous meta-analyses and systematic reviews while providing additional evidence-based support for optimizing fall risk reduction strategies in older adults [6–8, 14]*.* Both types emphasize center-of-gravity control and coordination between upper/lower body movements, thereby enhancing upper-body flexibility, agility, and balance proficiency in older adults [74]. MBE is highly suitable for older adults based on their physiological characteristics, and its ability to reduce the risk of falls has also been evidenced in several studies [75].

The results of this study found that MCPA with a longer duration is recommended to improve stability in older adults. Exercise-induced physiological adaptations demonstrate modality specificity: endurance training elevates maximal oxygen uptake through enhanced capillarization and aerobic enzyme activity, whereas resistance training augments force output via improved motor unit recruitment and hypertrophy [76]. Thus, evidence demonstrates that MCPA enhances neuroplasticity, improving older adults'cognitive function (e.g., planning capacity, selective/sustained attention) and physical capacity (aerobic endurance, lower-body strength, agility, balance/gait), with functional gains translating to daily living activities [77, 78]**.** It should be noticed, however, that exercise acceptance and adherence may be improved by the integration of exercise into one’s daily routine in a family environment, although the amount and intensity of exercise cannot be guaranteed [79, 80]. Therefore, further studies should explore how exercise intensity and duration could be determined.

The study on the volume of exercise and balance ability in older adults found that the balance capacity of older adults required an intervention of at least 11–12 weeks to be improved effectively [81]. In addition, challenging balance and functional exercises three or more times a week for more than 12 weeks is recommended in *the guidelines* for older adults to prevent falls [14]. This study demonstrates that the dynamic balance ability of older adults can be improved effectively by resistance exercise for 12–24 weeks, at least three times a week, for about 30–45 min. The findings from the Berg scale subgroup analysis indicated that the shorter exercise intervention period with MCPA had significant efficacy in the intervention of the capacity of the scale. Primarily, this may be because the Berg scale test is slightly easier to complete for older adults, as only a short exercise intervention period is required to improve ability. One review article on fall risk screening instruments suggests that older adults had generally high scores in the Berg scale test due to a ceiling effect [82]. Despite limitations in assessing balance improvements in high-functioning older adults, the scale remains a valid screening tool for fall risk. Future studies should further develop quantitative screening tools and establish specific cut-off values. The marked Intergroup variability in physiological profiles among older adults might constitute a key source of heterogeneity, as evidenced by subgroup analyses revealing maximal I^2^ = 93.5% when comparing studies [52, 65] with different average ages (about ten years difference) and baseline capacities of the experimental group (e.g., The unipedal stance test with eyes open, experimental group: 28.09 vs. 11.5 s; control group: 27.13 vs.16.3 s). In addition, meta-regression analysis identified that intervention session time might as a significant contributor to heterogeneity (*p* < 0.05). Our study was based on *the guidelines* emphasizing Predictive, Preventative, Personalised, and Participatory (4P) principles while providing empirical support for standardizing interventions through the Grading of Recommendations, Assessment, and Evaluation framework's evidence-grading architecture.

### Lower-extremity muscle strength and physical activity

Resistance training is the only known non-pharmacological intervention that is capable of counteracting the loss of bone and skeletal muscle, muscular strength, and speed qualities due to aging [83]. There is growing evidence suggesting that resistance exercise with relatively brief sessions can enhance the timeliness of exercise and improve muscle strength-related indices, leading to further improvements in independence and quality of life among older adults compared with conventional resistance exercise with greater total volume but poor engagement and adherence [83].

Exercises for fall prevention should provide a challenge to balance, which is beneficial for both improving balance ability and reducing the risk of falls [12]. Hewitt et al. [43] found that progressive resistance training at moderate intensities and high-challenge balance training significantly improved physical functioning and decreased the fall risk of older adults in senior care facilities. Our study findings are generally consistent with results from previous studies, from which we can identify specific intervention protocols. According to the schemes adopted in the included studies and the potential need for a longer recovery period in the elderly, the effectiveness of lower-extremity muscle strength and physical activity for older adults can be better improved with exercise interventions two times a week, for over 45 min of exercise each time, and a total duration of more than 12 weeks.

Despite different interventions and times of exercise improving lower-extremity muscle strength in older adults, differences were found in the optimal exercise regimes based on the pooled effect sizes of five stands sit-to-stand and the 30-s chair-stand tests. The five stands sit-to-stand among healthy seniors can be completed in about 10 s, whereas the 30-s chair-stand tests need to be performed with as many repetitions as possible in a 30-s time period. Differences in muscle type and energy metabolism processes may be observed in older adults due to different completion times for the tests. With increases in age, there is a reduction in myofiber diameter and total number, and the internal arrangement of skeletal muscle changes, in which type II muscle fibers become increasingly susceptible to the effects of age than type I muscle fibers [84]. Moreover, exercise intervention regimes for older adults should include increased exercise intensity and duration over 12–24 weeks, with low-intensity exercise to increase the effects of resistance exercise on the function of the myofiber contractile protein [76], which was consistent with our study. Moreover, based on these results, increasing frequency, extending the cycle, and shortening the time with MCPA might be more beneficial to further improve the ability to rapidly generate force. Thus, the formulation of an exercise regime should consider both the training principle of progressive overload and age-related degenerative changes in the musculoskeletal system (e.g., muscle fiber and motor neurons decrease) to allow adequate amount of time for recovery and adaptation and to prevent excessive fatigue [81].

### Mental efficacy

FES-I is used to understand the degree of concern about participating in simple or complex physical and social activities without falling. The “expected fear of falls” phenomenon is very common in community-dwelling elderly populations, which results in a decrease in balance, activity level, social participation, and quality of life [85]. Mental efficacy in older adults is influenced by balance problem, fall experiences, mood/temperament modulation, and emerging evidence highlighting cognitive factors—particularly attentional processing of sensory inputs [86]. This study suggests that meditation, exercise intervention, or physical perception practice reduces the fear of falling through exercise in older adults [87]. A previous study and meta-analysis found that exercise interventions, such as supervised functional exercises or a combination of strength and balance training, have a favorable effect on mental efficacy in older adults [14, 88]. Among them, MBE enhances psychosomatic coordination and somatic awareness through controlled movement sequences emphasizing the integration of cognitive, motor, and behavioral domains, while fostering a self-contemplative mental state [25, 26]. This mechanism confers superior efficacy in regulating mood compared to conventional physical training [89]. Findings by Donatoni et al. [36] demonstrated an increase in the heterogeneity of the MBE subgroup. Learning effects for the clinical assessment and the Hawthorne effect [90] may be responsible for the increased heterogeneity. Among many MBE, older adults may prefer traditional Chinese exercises, such as Tai Chi, as the primary modality of exercise, which can improve concentration and attention, help the individual to stay calm and relaxed in cases of strain and, importantly, increase balance and reduce the fear of falling while practicing soothing movements [45]. Furthermore, Tai Chi facilitates social interaction among older adults, which enhances self-efficacy and class attendance, thereby increasing the health benefits (e.g., balance, fall prevention, and psychosocial health) derived from sustained Tai Chi [91].

Results from this study suggest that an exercise period between 8 and 12 weeks may have positive effects on mental efficacy. However, Timon et al. [66] suggest that resistance exercise under moderate-to-low oxygen conditions for 45 min, three times per week, for 24 weeks reduces the fear of falling and improves health and physical fitness. This may result from different adherence rates (Group adherence to training was set at 75% attendance in the study [66]) or prescription variables when designing interventions due to the heterogeneity between physical functions in older adults. Another study recommended that exercise regimes should be formulated according to physical function levels in older adults, and the authors also highlighted the importance of consistent adherence to physical exercise [92].

This study synthesizes evidence on exercise interventions for fall risk reduction in community-dwelling older adults from a diverse range of countries, specifically examining the dose–response relationship between exercise prescription variables, thereby generating generalizable evidence-based recommendations for targeted intervention design. Per the Cochrane Handbook, the included studies demonstrated higher methodological quality. However, several limitations must be acknowledged. First, persistent publication bias was observed despite the trim and fill method application to verify robustness and in favor of the intervention condition. Second, the high heterogeneity observed across studies (I^2^ values ranging from 53.01% to 93.5%) suggests caution when generalizing the results. This variability may be due to differences in study design, population characteristics, and intervention specifics. Finally, critical analysis of exercise intensity parameters was precluded as the majority of included studies inadequately reported this prescription component. Future studies should aim to standardize intervention protocols and include more detailed reporting of exercise intensity and adherence. In addition, future studies should investigate the long-term efficacy and clinical effectiveness of these interventions to ascertain their practical benefits and sustained maintenance beyond the initial training period.

## Conclusions

This meta-analysis demonstrates that exercise interventions, particularly MBEs (e.g., Tai Chi) and MCPA, are effective in reducing fall risk by improving balance, muscle strength, and fall-related self-efficacy in older adults. In terms of practical application, the findings suggest that MCPA and MBE, such as Tai Chi, should be prioritized for older adults and integrated based on individual needs, particularly with durations of more than 8 weeks and each session lasting for over 30 min. These programs can be integrated into community health initiatives and tailored to individual needs to optimize balance and reduce the risk of falls. Tailored exercise programs, combining resistance exercises with balance training, should be designed to meet the individual needs of older adults, promoting both fall prevention and functional mobility.

## Data Availability

No datasets were generated or analysed during the current study.

## Abbreviations

CI: Confidence Interval
MBE: Mind–body Exercise
MCPA: Multi-component Physical Activity
MCID: Minimal Clinically Important Difference
MSA: Muscle-strengthening Activity
